# Antioxidant and **α**-Amylase Inhibitory Property of *Phyllanthus virgatus* L.: An *In Vitro* and Molecular Interaction Study

**DOI:** 10.1155/2013/729393

**Published:** 2013-06-19

**Authors:** Arshya Hashim, M. Salman Khan, Mohd. Sajid Khan, Mohd. Hassan Baig, Saheem Ahmad

**Affiliations:** ^1^Clinical Biochemistry and Natural Product Research Lab, Department of Biosciences, Integral University, Lucknow 226026, India; ^2^Department of Surgery, Cardinal Bernardin Cancer Center, Loyola University Medical Center, Maywood, IL 60153, USA

## Abstract

The present study on *Phyllanthus virgatus*, known traditionally for its remedial potential, for the first time provides descriptions of the antioxidant and inhibition of **α**-amylase enzyme activity first by *in vitro* analyses, followed by a confirmatory *in silico* study to create a stronger biochemical rationale. Our results illustrated that *P. virgatus* methanol extract exhibited strong antioxidant and oxidative DNA damage protective activity than other extracts, which was well correlated with its total phenolic content. In addition, *P. virgatus* methanol extract strongly inhibited the **α**-amylase activity (IC_50_ 33.20 ± 0.556 **μ**g/mL), in a noncompetitive manner, than acarbose (IC_50_ 76.88 ± 0.277 **μ**g/mL), which showed competitive inhibition. Moreover, this extract stimulated the glucose uptake activity in 3T3-L1 cells and also showed a good correlation between antioxidant and **α**-amylase activities. The molecular docking studies of the major bioactive compounds (9,12-octadecadienoic acid, asarone, 11-octadecenoic acid, and acrylic acid) revealed via GC-MS analysis from this extract mechanistically suggested that the inhibitory property may be due to the synergistic effect of these bioactive compounds. These results provide substantial basis for the future use of *P. virgatus* methanol extract and its bioactive compound in *in vivo* system for the treatment and management of diabetes as well as in the related condition of oxidative stress.

## 1. Introduction

Oxidative stress induced by reactive oxygen species (ROS) can cause cell membrane disintegration, protein, lipid, and deoxyribose nucleic acid (DNA) damage which can further initiate or propagate the development of many chronic and degenerative diseases [1–3]. When there is imbalance between ROS generation and antioxidant protection mechanism, it leads to cellular dysfunction causing various diseases inducing diabetes mellitus (DM) [4, 5]. Diabetes is an important metabolic syndrome affecting about 200 million people worldwide. The critical effect of diabetes is postprandial hyperglycemia and reduction in antioxidant defense mechanism. So, the management of type 2 DM could be done both by reducing oxidative stress as well as by delaying the absorption of glucose through the inhibition of any one of the carbohydrates-hydrolyzing enzymes, *α*-glucosidase, and *α*-amylase that are responsible for the breakdown of oligosaccharides and disaccharides into monosaccharides suitable for absorption [6–8].

There has been enormous interest in natural antioxidants due to their ability to neutralize the effects of ROS that are not only responsible for alleviating the oxidative stress condition in diabetes but are also helpful in managing the postprandial hyperglycemia. The growing interest to combat the side effect of the drugs available for diabetes leads to the development of green medicines due to their higher stability, higher antioxidant potential, low cost, and low cytotoxicity. Plants are rich sources of phytochemicals, which possess a variety of biological activities including antioxidant and antidiabetic potential both *in vitro* and *in vivo* [7–12].

In the last few decades, plants of genus *Phyllanthus *(Euphorbiaceae) came in focus due to their wide distribution, diversity in the genus, broad therapeutic potential, and variety in the secondary metabolites [13]. This family includes several plant species among all the species; *P. amarus*, *P. urinaria*, *P. maderaspatensis, P. virgatus,* and *P. fraternus *are the most popular ones due to their antioxidant properties as well as their extensive use in the treatment of disease related to kidney, liver, urinary bladder, intestinal infection, cancer, and diabetes [13–18].

It has been previously reported that *P. virgatus* is rich in polyphenols [13] and is known traditionally for its antioxidant [14], antimicrobial, antiseptic, anti-inflammatory agent [19], and anticancer activity [20]. The antidiabetic properties of various *Phyllanthus* species have been investigated in experimental models [15, 21]. However, only one study speculated the antidiabetic property of *P. virgatus* [21], and still the detailed investigation pertaining to their mechanism of action is lacking. So, this study was the first integrative approach to investigate and correlate the antioxidant, oxidative DNA damage protective activity, *α*-amylase inhibitory and glucose uptake property of various extracts of *P. virgatus*. Moreover, the mechanistic aspect of these compounds, elucidated via GC-MS analysis, was explored by carrying out docking studies against porcine pancreatic *α*-amylase in an array to give molecular mechanism of action of such inhibitors.

## 2. Methods

### 2.1. Chemicals

 Chemicals such as 1,1-diphenyl-2-picryl-hydrazyl (DPPH), 2,4,6-tripyridyl-s-triazine (TPTZ), ascorbic acid, thiobarbituric acid (TBA), Folin-Ciocalteu reagent (FCR), dimethyl sulfoxide (DMSO), dinitro salicylic acid (DNS), Dulbecco's modified Eagle medium (DMEM), dexamethasone (DEX), isobutylmethylxanthine (IBMX), foetal bovine serum (FBS), and 3-[4,5-dimethylthiazol-2-yl]-2,5-diphenyl tetrazolium bromide (MTT) were purchased from HiMedia Laboratories, Mumbai, India. Porcine pancreatic *α*-amylase was procured from SRL Pvt. Ltd., Mumbai, India. Methanol (MeOH), acetone, dichloromethane (DCM), *n*-hexane (*n*-hex), ethyl acetate (EtOAc), pUC18 plasmid, and Merckotest GOD/POD kit were obtained from Merck, India. Acarbose was obtained from Bayer Pharmaceuticals, and actrapid insulin was purchased from Torrent Pharmaceuticals Ltd., India. All chemicals were of analytical grade.

### 2.2. Collection and Preparation of Plant Extract

*P. virgatus* whole plant was collected from the local area around Integral University, Lucknow, India, in the months of July-August. The plant was botanically identified and authenticated by Dr. Mohd. Tariq, National Botanical Research Institute, Lucknow, India, and a voucher specimen (98195) of the plant was submitted there. *P. virgatus* whole plants were shed dried and made in coarse powder, avoiding sun dried due to the signature modification of the biochemicals. The dried powder (25 g) of the plants was extracted using nonpolar, partially polar, and polar solvents successively with the required amount of each of *n*-hex, DCM, EtOAc, MeOH, and water solvents in soxhlet apparatus until it turned colorless. The solvent was removed, filtered, and dried at room temperature, and residues were scratched out and stored at −20°C for future use. The percentage yield of different fractions was calculated by using the formula

(1) $$\begin{array}{c} \%\;\text{yield}=\frac{\text{weight of crude extract}}{\text{weight of raw material}\;}\times 100\text{.} \end{array}$$

The percentage yield was found to be *n*-hex: 2.17%, DCM: 0.75%, EtOAc: 0.37%, MeOH: 6.71%, and water: 2.31%.

### 2.3. Phytochemical Screening and Estimation of Total Phenol Content

Qualitative chemical tests were carried out to identify the phytochemicals present in various extracts of *P. virgatus* using standard procedure [22]. Total phenol content (TPC) of the extracts was determined by using Folin-Ciocalteu method [23].

### 2.4. DPPH Radical Scavenging Activity

The DPPH radical scavenging capacity of the various extracts of *P. virgatus* was determined by the method of Brand-Williams et al. [24]. Ascorbic acid was used as a reference standard. Percent (%) scavenging of DPPH free radical was measured using the following equation:

(2) $$\begin{array}{c} \%\;\text{DPPH}\;\text{radical}\;\;\text{scavenging}\\ \;=\left[\frac{\left(\text{absorbance}\;\text{of}\;\text{control}-\text{absorbance}\;\text{of}\;\text{test}\;\text{sample}\right)}{\left(\text{absorbance}\;\text{of}\;\text{control}\right)}\right]\\ \;\times 100\text{.} \end{array}$$

Further, IC_50_ value represented the concentration of the extract that caused 50% inhibition of DPPH radicals and was calculated by interpolation of linear regression analysis.

### 2.5. Ferric Reducing Antioxidant Potential

A modified method of Benzie and Strain [25] was adopted to determine the ferric reducing antioxidant potential (FRAP) of various extracts of *P. virgatus*. Briefly, the FRAP reagent was freshly prepared by mixing sodium acetate buffer (300 mM, pH 3.6), 10 mM TPTZ solution (in 40 mM HCl), and 20 mM Fe(III) chloride solution in a volume ratio of 10 : 1 : 1, respectively. Hundred microliters of the sample at various concentrations was added to 3 mL of the FRAP reagent. The absorbance was measured after 30 min at room temperature at 593 nm. The standard curve was plotted using FeSO_4_ solution, and results were expressed as *µ*mole Fe(II)/g dry weight of plant material.

### 2.6. Hydroxyl Radical Scavenging Assay

Hydroxyl radical scavenging activity of various extracts of *P. virgatus* was evaluated by the method of Badami et al. [26]. The percentage of hydroxyl radical scavenging potential was calculated by using the following formula, and IC_50_ was calculated as described previously:

(3) $$\begin{array}{c} \%\;\text{Hydroxyl}\;\text{radical}\;\text{scavenging}\;\text{activity}\\ =\left[\frac{\left(\text{absorbance}\;\text{of}\;\text{control}-\text{absorbance}\;\text{of}\;\text{test}\;\text{sample}\right)}{\left(\text{absorbance}\;\text{of}\;\text{control}\right)}\right]\\ \;\times 100\text{.} \end{array}$$

### 2.7. DNA Protection Assay

DNA protection assay was performed using supercoiled pUC18 (2686 bp) plasmid DNA according to the method of Lee et al. [27] with slight modifications. Plasmid DNA (250 ng) was incubated with Fenton's reagent containing H_2_O_2_ (30 mM), ascorbic acid (100 *µ*M), and FeCl_3_ (160 *µ*M) in the presence and absence of the plant extract, and the final volume of the mixture was raised up to 20 *μ*L. The mixture was then incubated for 45 min at 37°C followed by the addition of loading dye, and the electrophoresis was carried out in Tris-acetate-EDTA buffer (40 mM Tris base, 16 mM acetic acid, and 1 mM EDTA; pH 8.0). DNA was analyzed followed by ethidium bromide staining, and mannitol was used as positive control.

### 2.8. *α*-Amylase Inhibition Assay

To determine the *in vitro* *α*-amylase inhibition by various extracts of* P. virgatus,* the standard procedure [28] was adopted with slight modification. Briefly, porcine pancreatic *α*-amylase was dissolved in ice-cold phosphate buffer (20 mM), pH 6.7, containing sodium chloride (6.7 mM) to give a concentration of 0.15 unit/mL. Triplicate test tubes including the blank were prepared. In each test tube, 250 *μ*L of the enzyme preparation was mixed with 100 *μ*L of each of the extracts except the blank. The mixtures were stirred in a vortex and preincubated in a water bath at 37°C for 20 minutes. After incubation, 250 *μ*L of the substrate preparation (0.5% w/v starch in 20 mM phosphate buffer; pH 6.7) was transferred into each test tube to start the reaction. The mixture was vortexed and then incubated at 37°C for 15 minutes. Two mL of DNS color reagent (DNS 40 mM, K-Na tartrate 1 M, and sodium hydroxide 0.4 M) was added, vortexed and boiled in a water bath at 100°C for 10 minutes. Thereafter, the mixture was cooled down, and the absorbance was read at 540 nm. Acarbose was used as standard inhibitor.

Inhibition rates were calculated as percentage controls using the formula:

(4) $$\begin{array}{c} \%\;\text{inhibition}=100-\%\;\text{reaction}, \end{array}$$

where % reaction = (mean product in sample/mean product in control) × 100. 

Further, IC_50_ value represented the concentration of the extract that caused 50% inhibition of *α*-amylase and was calculated by interpolation of linear regression analysis.

### 2.9. Determination of Mode of Inhibition

Mode of inhibition of* P. virgatus* methanol extract against *α*-amylase was determined by the method of Mogale et al. [29]. For the assay, two sets (A and B) of 6 duplicate test tubes were prepared to determine the enzyme activity in the presence [set A] and absence [set B] of an inhibitor (methanol extract/standard acarbose). In set A, 100 *μ*L of the inhibitor (plant extract or acarbose, 1 mg/mL) solution was added in each test tube except the blank; this was followed by the addition of 100 *μ*L of the enzyme porcine *α*-amylase (0.15 units/mL). In set B, 100 *μ*L of phosphate buffer (20 mM), pH 6.7, containing sodium chloride (6.7 mM) was added in each test tube followed by 100 *μ*L of the enzyme solution. Both sets of test tubes were thoroughly mixed in a vortex mixer and preincubated in a water bath at 37°C for 20 minutes. Serial dilutions of the substrate solution were added in both sets of test tubes with concentration ranging between 2.5 *µ*g/mL and 0.156 *µ*g/mL. All the tubes were then incubated at 37°C for 15 minutes, followed by the addition of 2 mL of DNS color reagent and the mixtures were boiled for 10 minutes. Absorbance of the colored solution was read at 540 nm. Double reciprocal curve (1/*V v*/*s* 1/[*S*]) for both sets was plotted to determine the effect of the plant extract/acarbose on *V*_max⁡_ and *K*_*m*_ of the enzyme, where *V* and [*S*] are, respectively, the velocity of the reaction and substrate concentration.

### 2.10. Cell Culture

3T3-L1 preadipocytes cell lines are known to mimic *in vivo *organs that have an influence on glucose homeostasis. These cell lines were obtained from National Centre for Cell Sciences (NCCS), Pune, India. Cells were routinely cultured at 37°C in a humidified 5% CO_2_, 95% air atmosphere and were grown in DMEM medium supplemented with 10% FBS, L-glutamine (8 mM), and 2% antimycotic. 

### 2.11. Cell Viability Assay

The standard MTT colorimetric assay [30], which is based on the reduction of MTT by mitochondrial dehydrogenase to a purple formazan product, was used to assess the cytotoxic activity of *P. virgatus* methanol extract. The effect was quantified as follows:

(5) $$\begin{array}{c} \%\;\text{inhibition}\\ \;=\frac{\text{absorbance}\;\text{of}\;\text{the}\;\text{control}-\text{absorbance}\;\text{of}\;\text{sample}}{\text{absorbance}\;\text{of}\;\text{control}}\\ \;\times 100\text{.} \end{array}$$

### 2.12. Glucose Uptake Assay

Cells were cultured and plated at a density of 12,000 cells/well in a 24-well plate and incubated for 24 hours in the DMEM growth media containing 5 mM glucose. On day 1, the growth medium was replaced by supplemented medium, which consisted of DMEM supplemented with 10% FCS, insulin (10 *μ*g/mL), DEX (10^−8^ M), and IBMX (0.1 mM). Cells were refed 48 hours later with the same supplemented medium, and after another 24 hours (day 4), this medium was removed and replaced with growth medium including the treatment protocol [11] (Table 1). After a further 48-hour incubation (day 6), the cells were assayed in their appropriate experiments. Ten microliters of the media was removed and placed in the 96-well plates to which 200 *µ*L of GOD/POD reagent was added and incubated for 15 min at 37°C. The change in the color was recorded at 495 nm. The following equation was used to calculate the glucose content (mg/dL) in each well. Concentration of unknown sample = (concentration of sample/abs. of standard − abs. of reagent blank) × abs. of unknown sample − abs. of reagent blank). Finally, glucose uptake over control was calculated as the difference between the initial and final glucose content in the incubated medium.

### 2.13. Gas Chromatography and Mass Spectroscopy (GC-MS) Analysis

In order to know the bioactive metabolites responsible for antioxidant and antidiabetic activity, the methanol extract of *P. virgatus* was subjected to GC-MS analysis. The sample was injected into an RTX-5 column (60 m × 0.25 mm i.d., film thickness 0.25 *µ*m) of GC-MS (model GC-MS-QP-2010 plus, Shimadzu Make). Helium was used as carrier gas at a constant column flow of 1.2 mL/min at 173 kPa inlet pressure. Temperature programming was maintained from 100°C to 200°C with constant rise of 5°C/min and then held isothermal at 200°C for 6 min; further, the temperature was increased by 10°C/min up to 290°C and again held isothermal at 290°C for 10 min. The injector and ion source temperatures were 270°C and 250°C, respectively. Mass spectra were taken at 70 eV a scan interval of 0.5 s and fragments from 40 to 950 Dalton. The final confirmation of constituents was made by computer matching of the mass spectra of peaks with the National Institute of Standards and Technology (NIST) libraries mass spectral database.

### 2.14. Docking Analyses

#### 2.14.1. Preparation of Enzyme and Ligand

The crystal structure of porcine pancreatic *α*-amylase (PDB ID: 1DHK) in complex with acarbose was retrieved from Research Collaboratory for Structural Bioinformatics (RCSB) protein databank. Water molecules as well as other heteroatoms were removed, and the protein was subjected to energy minimization using CharMM force field [31]. The method deployed for energy minimization was steepest descent at RMS gradient of 0.1 for 1000 steps. Compounds, whose 3-D structures were available, were extracted from PubChem compounds database, while those whose structures were not present were drawn using ChemDraw like hexadecanoic acid, 9,12-octadecadienoic acid, 11-octadecenoic acid, and 6-octadecynoic acid. 

#### 2.14.2. Molecular Docking

Molecular docking studies were carried out using AutoDock program [32] to get the favorable binding modes for compounds within the active site of porcine pancreatic *α*-amylase. Before conducting molecular docking, validation was performed, in which the acarbose present within the binding site of porcine *α*-amylase in crystal structure was extracted. This acarbose was subjected to redocking within the active site of porcine *α*-amylase using AutoDock. The binding confirmation was visualized using PyMOL. After complete execution of AutoDock, various conformations of ligand in complex with the receptor were obtained, which were finally ranked on the basis of binding energy. 

### 2.15. Statistical Analysis

The results were analyzed by using one-way analysis of variance (ANOVA) and two-tailed Student's *t*-test. Statistical significance was expressed as *P < 0.05,  **P < 0.01, and ***P > 0.001.

## 3. Results

### 3.1. Phytochemical Estimation and Total Phenol Content

Our results illustrated significant presence of tannins, terpenoids, saponins, phenols, carbohydrate, flavanoids, protein, glucose, and reducing sugar in *P. virgatus* methanol extract (Table 2). Water extract contains all the above phytochemicals except glucose and reducing sugar. In addition, EtOAc extract contains terpenoids, flavanoid, protein, glucose, and reducing sugar, while only tannins, terpenoids, and protein were present in DCM extracts. In contrast, *n*-hex contains only protein content. The TPC (*µ*g/mg GAE) in the various extracts of *P. virgatus* was also determined and found to be in the following decreasing order: MeOH > water > EtOAc >  *n*-hex > DCM. From the data, it is evident that methanol extract has higher phenolic content (176.68 ± 0.032 *µ*g GA/mg plant extract) than the water and ethyl acetate extracts, whereas DCM and *n*-hex have the lowest phenolic content (Table 3). 

### 3.2. Total Antioxidant Activity

**Figure 1:**
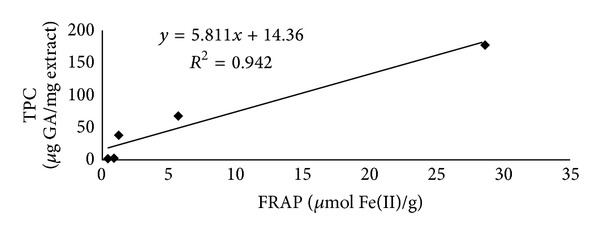
Linear correlation between the amount of TPC and antioxidant capacity (FRAP) of *P. virgatus* in various solvent systems.

Antioxidant activities of different *P. virgatus* extracts were assessed by FRAP assay, which is based on their ability to reduce ferric ions to ferrous form. The results illustrated that methanol extract has significantly higher FRAP values (28.61 ± 0.2184 *μ*mol Fe(II)/g) as compared to other extracts (0.4–6 *µ*mol Fe(II)/g) and standard ascorbic acid (Table 3). A simple linear regression analysis was used to analyze the correlation between FRAP value and the TPC.  Figure 1 showed linear corelation between the total phenols and the FRAP value. The coefficient of determination was 0.942 between the TPC and the antioxidant (FRAP) value, indicating that the antioxidant capacity of the extracts can be attributed to their phenolic compounds.

### 3.3. DPPH Radical Scavenging Activity

**Figure 2:**
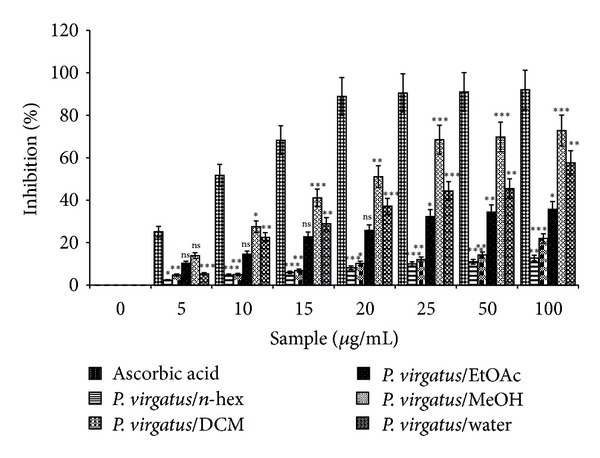
DPPH radical scavenging activity of different extracts of *P. virgatus* and standard ascorbic acid. The data represent percent scavenging of DPPH radicals. The results are mean ± S.D. of three parallel measurements. Nonsignificant (ns), *P < 0.05, ^ ∗∗^ *P* < 0.01,  ***P < 0.001 versus 0 *μ*g/mL.

The relatively stable DPPH radical is widely used to evaluate the free radical scavenging activity of various natural antioxidants including plant extracts. The data present in Figure 2 showed the percent inhibition of DPPH radical scavenging activity of different extracts of *P. virgatus*. The methanol fraction of *P. virgatus* exhibited higher antioxidant activity with an IC_50_ value of 18.59 ± 0.515 *µ*g/mL as compared to ascorbic acid (Table 4). IC_50_ value of EtOAc, DCM, and *n*-hex extract failed to show any significant scavenging activity, whereas the water extract showed an IC_50_ value of 40.36 ± 2.35 *µ*g/mL. From the data, we observed that DPPH radical scavenging activity was increased as the concentration increased for each individual extract, with marked increase in methanol extract (Figure 2). 

### 3.4. Hydroxyl Radical Scavenging and Oxidative DNA Damage Protective Activity

**Figure 3:**
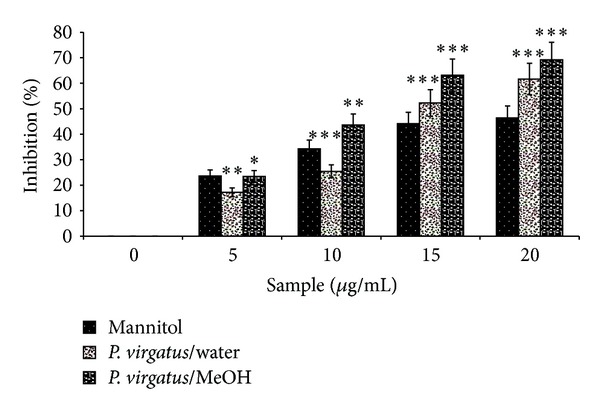
Hydroxyl radical scavenging activity of the *P. virgatus *MeOH, water extract, and reference compound mannitol. The data represents the percentage of inhibition of deoxyribose degradation. The results are expressed as mean ± S.D. (*n* = 3). *P < 0.05,  **P < 0.01,  ***P < 0.001 versus 0 *μ*g/mL.

Hydroxyl radicals (OH^∙^) are singlet oxygen species, which are highly reactive and cause damage to various biological macromolecules. Therefore, the role of different extracts of *P. virgatus* in directly scavenging and in protecting the DNA damage, caused by hydroxyl radical, was evaluated. The data presented in Figure 3 clearly indicates better scavenging activity of the methanol extract with an IC_50_ value of 12.53 ± 2.38 *μ*g/mL than water extract, while the other extracts (data not shown) including mannitol showed insignificant scavenging activity of hydroxyl radical.

**Figure 4:**
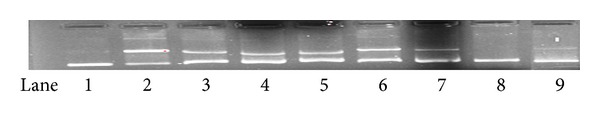
Effect of *P. virgatus* water and MeOH extracts on damaged supercoiled pUC18 plasmid DNA. Lane 1: pUC18 DNA + PBS; lane 2: pUC18 DNA + Fenton's reagent; lane 3: DNA + Fenton's reagent + water extract (50 *μ*g/mL); lane 4: DNA + Fenton's reagent + water extract (100 *μ*g/mL); lane 5: DNA + Fenton's reagent + water extract (200 *μ*g/mL); lane 6: DNA + Fenton's reagent + MeOH extract (50 *μ*g/mL); lane 7: DNA  + Fenton's reagent + MeOH extract (100 *μ*g/mL); lane 8: DNA + Fenton's reagent + MeOH extract (200 *μ*g/mL); lane 9: Mannitol (200 *μ*g/mL).

The oxidative DNA damage protective activity of *P. virgatus *methanol and water extracts showed almost complete and partial protection of OH^∙^-induced oxidatively damaged plasmid DNA, respectively (Figure 4). Incubation of pUC18 plasmid DNA with Fenton's reagent resulted in the cleavage of supercoiled form to give open circular and linear forms of plasmid DNA, indicating that OH generated from iron-mediated decomposition of H_2_O_2_ produced both single-strand and double-strand DNA breaks. Addition of *P. virgatus* methanol and water extracts (50,100 and 200 *µ*g/mL) showed complete and partial protection of supercoiled DNA (Figure 4). 

### 3.5. *α*-Amylase Inhibitory Property

**Figure 5:**
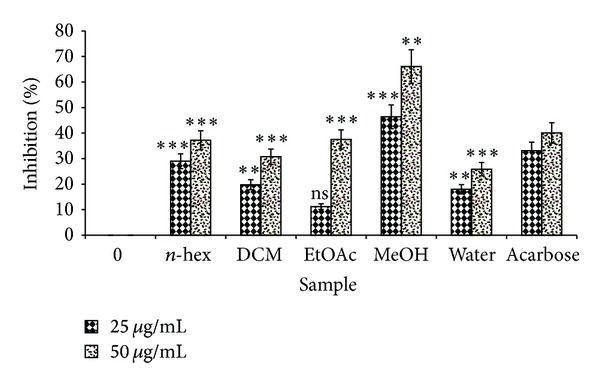
Screening of *α*-amylase inhibitory property of various extracts of *P. virgatus*. Results are mean ± S.D. of three parallel measurements. Nonsignificant (ns), **P < 0.01,  ***P < 0.001 versus 0 *μ*g/mL.

**Figure 6:**
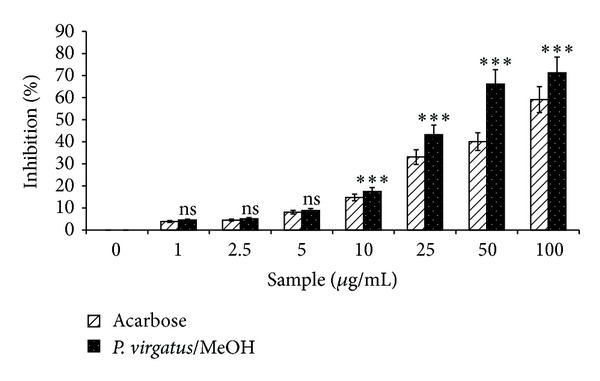
Concentration-dependent inhibition of *P. virgatus* methanol extract and reference compound acarbose. The results are expressed as mean ± S.D. of three parallel experiments. Nonsignificant (ns), ***P < 0.001 versus 0 *μ*g/mL.

In an array to explore the antidiabetic activity, various extracts of *P. virgatus* were screened for the *α*-amylase inhibitory property. Initial screening of various extracts showed that the methanol extract has significantly higher percentage of *α*-amylase inhibition, that is, 43.2% and 66.09% at 25 and 50 *µ*g/mL, respectively (Figure 5). Furthermore, the methanol extract showed concentration-dependent increase in percent inhibition of *α*-amylase activity and also exhibited a lower IC_50_ value (33.20 ± 0.556 *µ*g/mL) than standard drug acarbose, which in turn indicates a potent antidiabetic property of this extract (Figure 6; Table 4).

**Figure 7:**
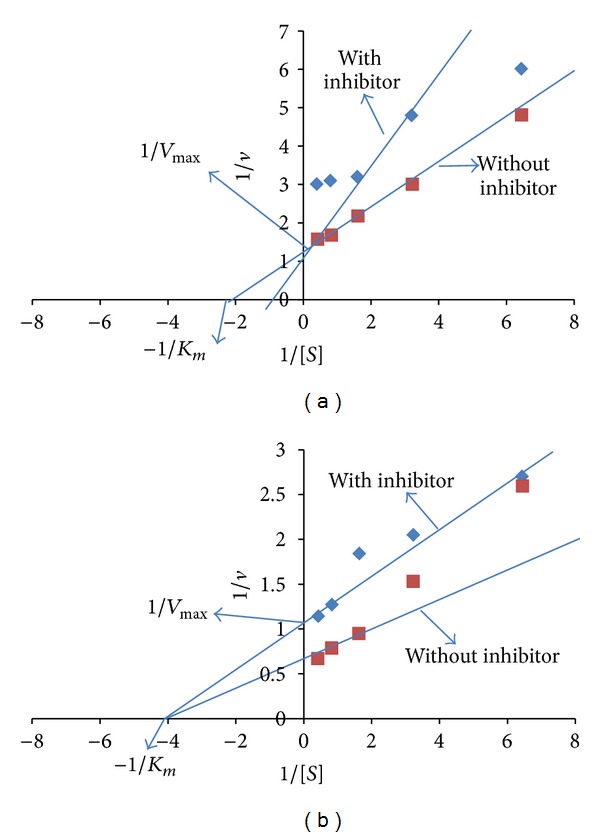
Lineweaver-Burk double reciprocal plot of 1/*v* versus 1/[*S*] of acarbose (a) and *P. virgatus* methanol extract (b) against *α*-amylase.

**Figure 8:**
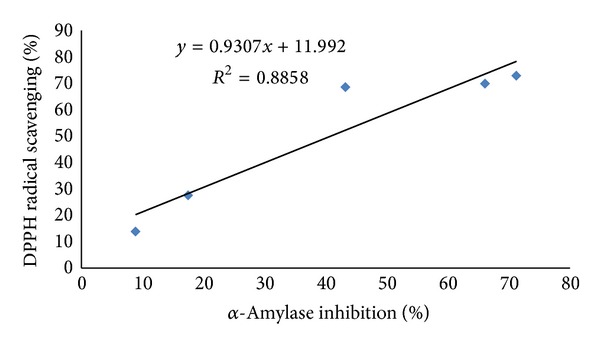
Linear correlation between the DPPH radical scavenging activity and *α*-amylase inhibition of *P. virgatus* methanol extract.

The mode of inhibition of acarbose and methanol extracts of *P. virgatus *against porcine *α*-amylase was also determined by means of Lineweaver-Burk double reciprocal plot of 1/*v* versus 1/[*S*]. The methanol extract showed a noncovalent type of noncompetitive inhibition against porcine *α*-amylase (Figure 7(b)), whereas acarbose was competitive in nature (Figure 7(a)). In addition, a linear correlation was also observed between DPPH radical scavenging activity and *α*-amylase inhibitory property of *P. virgatus* methanol extract (*R* ^2^ = 0.885) (Figure 8).

### 3.6. Glucose Uptake Assay

**Figure 9:**
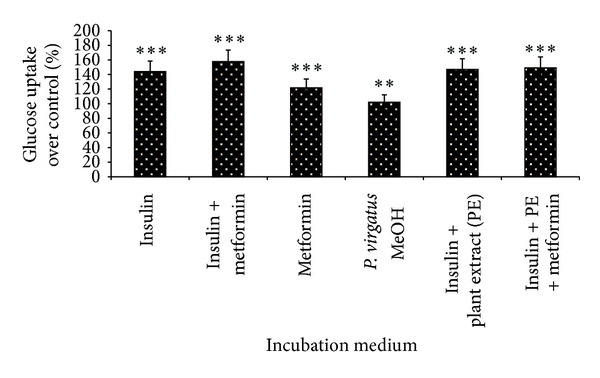
Effect of *P. virgatus *methanol extract on glucose utilization in differentiated 3T3-L1 cell line. Results are mean ± S.D. of three parallel measurements. **P < 0.01 and ***P < 0.001 indicate significance compared to unstimulated cells.

It was found that the methanol extract of *P. virgatus* showed strong *α*-amylase inhibitory property; further, its role in glucose utilization in differentiated 3T3-L1 adipocytes cell line was also studied *in vitro.* The ability of the plant extract to induce glucose uptake was tested in different combinations (Table 1), and it was found that the methanol extract of *P. virgatus* alone showed significant glucose utilization up to 102%; in the presence of insulin it increases up to 146.8%, and in the presence of both insulin and metformin, the glucose uptake of extract was markedly increased by 149% over the control value. In addition, insulin and metformin alone showed 144% and 122% of glucose uptake, while 158% of glucose uptake was observed during their synergistic treatment (Figure 9).

**Figure 10:**
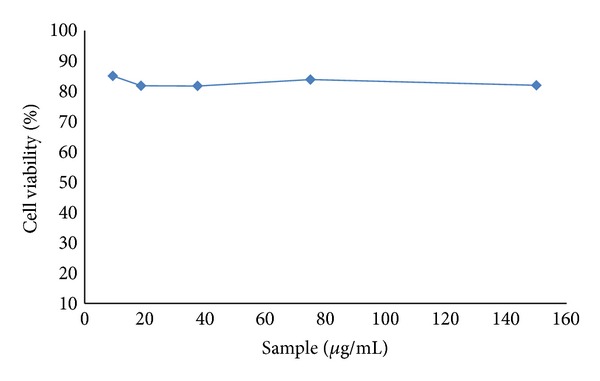
Percent of cell viability of 3T3-L1 adipocytes treated with methanol extract of *P. virgatus *at various concentrations.

Moreover, to verify the cytotoxicity of methanol extract, the MTT assay on 3T3-L1 preadipocytes cell line was studied *in vitro,* and it was found that the methanol extract of *P. virgatus* was noncytotoxic at various concentrations as shown in Figure 10. Since the presented data clearly indicates that methanol extract showed markedly higher antioxidant and *α*-amylase inhibitory property, it was further subjected to preliminary GC-MS analysis. 

### 3.7. GC-MS Analysis

Preliminary GC-MS analysis, based on retention time and molecular mass, was performed to determine the nature of phytoconstituents present in methanol extract. The GC-MS spectral results and comparison of results with library search successfully enabled the identification of seven compounds with their retention time (RT), molecular formula, molecular weight (MW), and concentration (peak area %) (Table 5). The results revealed that 2,4,5-trimethoxy propenylbenzene (27%), 11-octadecenoic acid (22.29%), 9,12-octadecadienoic acid (16.91%), and hexadecanoic acid (13.36%) were found to be the four major components in the methanol extract, whereas benzenedicarboxylic acid (7.1%), tridecyl ester (8.77%), and 6-octadecynoic acid (4.56%) were found in less amounts.

### 3.8. Docking Analyses

**Figure 11:**
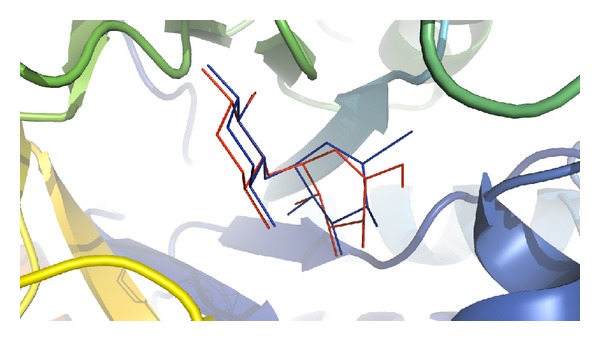
Binding orientation of the crystallized (red) and redocked (blue) acarbose.

**Figure 12:**
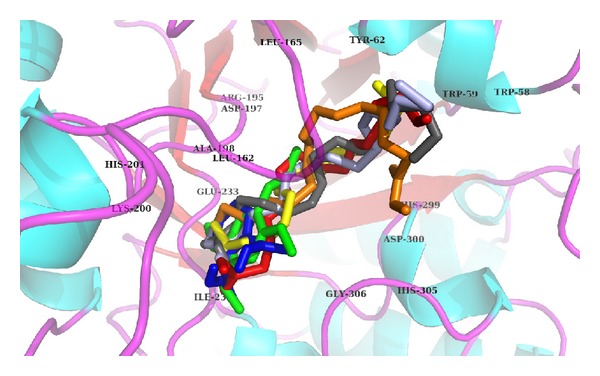
Binding pattern of the compounds depicted via GC-MS analyses within the active site of pancreatic *α*-amylase.

Validation of docking protocol and the size as well as center of the coordinates of the grid was carried out in order to ensure that ligands bind to the binding pocket in the correct conformation. It was performed by redocking cocrystallized acarbose into its respective binding site within porcine pancreatic *α*-amylase. Redocked inhibitor was found to interact with the same amino acids of the active site as was in the crystal structure (Figure 11). The root mean square deviation (RMSD) of all atoms between these two conformations was 1.58 Å, indicating that the protocol set for molecular docking is accurate. Then, the *in silico* study was done beneath the assumption that a predicted high docking score in absolute value will be predictive of a strong inhibition of the enzyme. The results showed that all the molecules depicted by GC-MS were found to bind within the active site of porcine pancreatic *α*-amylase with binding free energy ranging from −3.27 to −6.11 Kcal/mol (Table 6). The docking results illustrated that 9,12-octadecadienoic acid was the most active compound, followed by 11-octadecenoic acid, asarone, and acrylic acid with binding free energy of −6.11, −5.55, −5.21, and −5.19 Kcal/mol, respectively, against *α*-amylase. The following amino acids: Trp57, Tyr62, Leu162, Leu165, Asp197, Lys200, His201, Glu233, and Ile235 were found to be the key residues playing important role in stabilizing the complex (Figure 12 and Table 6).

## 4. Discussion

Currently used synthetic drugs, which are known to protect against type 2 DM and oxidative damage, have their adverse side effects. As a result, consumption of natural antioxidants, that are known to be effective scavengers of free radicals, through plants, food, or dietary supplements, interrupts the production of ROS and thus helping in the prevention of various diseases including type 2 DM [4, 8, 9, 33, 34]. In the current study, a sequential extraction involving the solvent of decreasing polarity to extract the bioactive compounds was used because the nature, polarity, and hence the solubility of the bioactive compounds in *P. virgatus* were unknown. Our data initially indicated that methanol extract of *P. virgatus* not only showed significant presence of polyphenols but also exhibited the highest amount of TPC than other extracts, which is in well agreement with earlier studies [14, 35]. Our results illustrated significantly higher total antioxidant capacity of the methanol extract of *P. virgatus*, which is even greater than the standard ascorbic acid. The reducing capacity of a compound may serve as a significant indicator of its potential antioxidant activity and generally correlates with the presence of reductones, which have been shown to exert antioxidant activity by breaking the free radical chain and donating a hydrogen atom [36].

Phenolic compounds have been said to account for most of the antioxidant activities of plant extracts [37], and thus antioxidant activity of methanol extract would be granted to these polyphenolic compounds. Our results observed high linear correlation between the FRAP value and TPC of various extracts of *P. virgatus*, indicating that the antioxidant activity of the extract is mainly due to its phenolic content.

It is well known that compounds capable of scavenging free radicals can delay, inhibit, or prevent the oxidation of various biomacromolecules and diminish the oxidative stress, which play major role in the development of several diseases like diabetes [4, 5, 10]. Our results demonstrated that *P. virgatus* methanol extract exhibited strong DPPH radical scavenging activity with respect to other extracts (Table 4), which in turn signifies its potent antioxidant activity. These results are in concordance with another study where crude methanol extract of *P. virgatus* showed higher DPPH radical scavenging activity [20]. 

A great number of *in vitro* experiments showed that hydroxyl radical, the most reactive among ROS, has the capacity to damage DNA, which appears to represent the major target involved in mutagenesis, carcinogenesis, diabetes, and so forth [3, 38]. Therefore, we initially evaluated the role of different extracts of *P. virgatus* in directly scavenging and in protecting the DNA damage caused by hydroxyl radicals. The degradation of deoxyribose to thiobarbituric acid reactive substances (TBARS) by hydroxyl radicals was markedly decreased by methanol and water extract. The observed IC_50_ value of *P. virgatus* methanol extract indicates that this extract is a better hydroxyl radical scavenger than standard mannitol. There are several reports indicating that various antioxidants present in plant are good scavengers of hydroxyl radicals [39], including only one report from *Phyllanthus* sp., that is maderaspatensis [17]. 

Damage of plasmid DNA due to OH^·^ radical resulted in single- and double-strand break. Studies have identified potent antioxidants from plants that are effective against DNA damage [38]. *P. virgatus* was also known for its potent antioxidant property [20], and our preliminary result showed that the DNA damage induced by hydroxyl radical was significantly ameliorated by *P. virgatus* methanol extract (Figure 4) and is almost comparable with standard mannitol. The above action of *P. virgatus* methanol extract was may be due to its strong antioxidant activity, which prevents the reaction of Fe^2+^ ions with H_2_O_2_ or through mechanism including quenching of ROS by donating H atom. The result illustrating the potent oxidative DNA damage protective activity of methanol extract is well correlated with results of our various antioxidant parameters, illustrating the greater ROS-quenching capacity by methanol extract, which in turn indicates that this extract may be used as therapeutic agent in treating ROS-related pathological conditions including type 2 DM. 

 There are several therapeutic approaches to decrease postprandial hyperglycemia; one of which is retarding the absorption of glucose through inhibition of carbohydrate-hydrolyzing enzymes either *α*-amylase or *α*-glucosidase [6–8]. Presently used oral antihyperglycemic agents have however a risk of inducing hypoglycemia and lose their efficacy over time, and they have prominent side effects and fail to significantly alter the course of diabetic complications [7]. The management of diabetes without any side effects is still a challenge; therefore, the World Health Organization (WHO) has recommended research and use of complementary medicines from plants for the treatment and management of this disease [40]. 

 Our investigation provides the first evidence of *α*-amylase inhibitory property of sequentially extracted *P. virgatus* extracts. The initial screening of all extracts of *P. virgatus* demonstrated some *α*-amylase inhibitory activity with maximum activity in methanol extract. In addition, this extract also exhibited a concentration-dependent increase in percent inhibition of *α*-amylase activity with an IC_50_ value of 33.20 ± 0.556 *μ*g/mL, which is quite better than the IC_50_ value of standard acarbose 76.88 ± 0.277 *μ*g/mL. This observation suggests that porcine pancreatic *α*-amylase is inhibited by more polar constituents of *P. virgatus*, which is in agreement with another study that reported *α*-amylase inhibitory activities in the more polar extracts of plant materials [41, 42]. Thus, the enzyme inhibitory activity of methanol extracts could be due to the presence of polyphenols, flavonoids, and their glycosides, which are known to be soluble in more polar solvents.

Enzymes inhibitors obeying Michaelis-Menten kinetics are often characterized in terms of their effects on the kinetic constants, *K*_*m*_ and *V*_max⁡_, using either Lineweaver-Burk plots or Dixon secondary plots. In the current study, *P. virgatus* methanol extracts demonstrated noncovalent type of noncompetitive (*V*_max⁡_ decreased whereas *K*_*m*_ remained the same) mode of inhibition against porcine pancreatic *α*-amylase, whereas acarbose was competitive in nature. These observations might suggest that the *α*-amylase inhibitory components of methanol extracts do not resemble the normal substrates of the enzymes in structure [29]. Further, the mechanism of inhibition of acarbose seems to be due to the unsaturated cyclohexene ring and the glycosidic nitrogen linkage that mimics the transition state for the enzymatic cleavage of glycosidic linkages [43]. It has been previously shown that acarbose is a competitive inhibitor of *α*-amylase, which is in strong agreement with our results [29]. 

Moreover, the strong correlation demonstrated between total antioxidant and polyphenol contents and between DPPH radical scavenging and *α*-amylase activity of methanol extracts suggests that polyphenol compounds involved in total antioxidant/antiradical activity may also is directly or indirectly intervene in *α*-amylase inhibitory activity. Postprandial blood glucose level is known to be regulated by glucose uptake, a rate limiting step for glucose metabolism. In the present study, we used differentiated 3T3-L1 cell lines because it was previously established that glucose uptake was higher in these cells than in undifferentiated one, which is probably due to the presence of glucose transporter-4 (GLUT4) in their expression [44]. 

Our result, for the first time, speculated that *P. virgatus *methanol extract possesses the ability to improve glucose uptake in the adipose tissue. The positive controls chosen for glucose uptake in 3T3-L1 cells due to their antidiabetic activity were metformin and insulin, as they are known specifically for the affirmative effect on the translocation of GLUT4 to the cell surface thereby promoting glucose uptake. Insulin and metformin significantly promote the glucose uptake alone, while there is almost a negligible synergistic effect when given in combination (in presence or absence of extract). On this basis, a mechanism of action of *P. virgatus *may be hypothesized, which could be linked to insulin-mediated glucose transport transduction pathway in which a series of proteins (phosphatidylinositol-3 kinase, protein kinase C, and PPAR) are involved [44, 45]. This may perhaps lead to the translocation of GLUT4 to the plasma membrane to facilitate the uptake of glucose from the bloodstream into the cells. Thus, the occurrence of polyphenolic compounds in methanol extract may be responsible for the activation of these signaling proteins [11, 46, 47] and might therefore also account for their upregulation of these proteins, which in turn is responsible for its glucose uptake activity. 

In a search for the source of bioactive compounds responsible for the aforementioned actions of *P. virgatus* methanol extract, preliminary GC-MS analysis was performed. For the first time, it was noted that the sequentially extracted *P. virgatus* methanol extract contains phthalic acid, asarone, acrylic acid, palmitic acid, linoleic acid, 11-octadecenoic acid, and 6-octadecynoic acid (Table 5). Various species of *Phyllanthus* reported the presence of these compounds with antioxidant and antidiabetic activity [13, 48, 49]. 

 From the above data, it may be concluded that these bioactive compounds of *P. virgatus* methanol extract alone or in combination possess significant antioxidant activity, which could be responsible in ameliorating all the above oxidative damages, including inhibition of amylase activity. However, our *in silico* investigation is a novel approach to identify the molecular targets involved in inhibition of *α*-amylase activity by this extract. We previously revealed the implication of molecular docking studies in elucidating the mechanistic aspect of natural products against different enzymes [50]. Molecular simulation study is considered to be an important vehicle to investigate the mode of interaction of ligand against its target protein that also makes us understand their binding or inhibition mechanism. Validation of docking protocol was performed by redocking cocrystallized acarbose into its respective binding site within porcine pancreatic *α*-amylase. Redocked inhibitor was found to interact with the same amino acids of the active site as was in the crystal structure with RMSD of 1.58 Å between these two conformations (Figure 11). Our results demonstrated that 9,12-octadecadienoic acid (linoleic acid) was the most potent inhibitor of pancreatic *α*-amylase, whereas 11-octadecenoic acid, 2,4,5-trimethoxy propenyl (asarone), and tridecyl ester also showed good inhibitory activity in terms of their binding energy. These results are well supported by wet lab studies where asarone, linoleic acid, and acrylic acid have been reported to exhibit the antidiabetic property [50, 51]. Of the catalytic triad [52], Asp197 and Glu233 were found to be very much involved in the positioning of inhibitor within the active sites of pancreatic *α*-amylase. Our results are in general agreement with the mechanism of action proposed for acarbose [8], since they showed for *α*-amylase that active ligands interact with the side chains of Asp197, Glu233, and Asp300 (Figure 12, Table 6). All the inhibitors were anchored at the catalytic center, which might explain why the enzymatic activity of *α*-amylase was successfully blocked. Thus, it is very difficult to name a single compound responsible for the whole activity. Therefore, based on our *in vitro* and *in silico* results, we suggest that the *α*-amylase inhibitory activity of *P. virgatus* methanol extract might be because of the synergistic effect of these compounds.

## 5. Conclusion

In conclusion, the results for the first time demonstrated a strong antioxidant and *α*-amylase inhibitory as well as glucose uptake property of sequentially extracted *P. virgatus* methanol fraction, compared to other extracts. The docking studies further confirmed the antidiabetic property of the bioactive compounds revealed via GC-MS analysis of this extract and suggested that *α*-amylase inhibitory property of this extract was maybe due to the synergistic effect of these bioactive compounds. Thus, it is a good approach to manage type 2 DM as a whole with these compounds/extracts, which showed good enzyme inhibitory and antioxidant activities. Further, a thorough and full-fledged *in vivo* study is needed to explore the role of these extracts and also their bioactive compounds.

## Figures and Tables

**Table 1:** Treatment protocol for glucose uptake assay.

S. no.	Incubation medium
Group 1	1000 *µ*L DMEM containing 5 mM glucose
Group 2	900 *µ*L DMEM + 100 *µ*L insulin (1 IU/mL)
Group 3	900 *µ*L DMEM + 100 *µ*L metformin (1 mg/1 mL)
Group 4	900 *µ*L DMEM + 100 *µ*L plant extract (1 mg/mL)
Group 5	800 *µ*L DMEM + 100 *µ*L insulin (1 IU/mL) + 100 *µ*L plant extract (1 mg/mL)
Group 6	800 *µ*L DMEM + 100 *µ*L insulin (1 IU/mL) + 100 *µ*L metformin (1 mg/1 mL)
Group 7	700 *µ*L DMEM + 100 *µ*L insulin (1 IU/mL) + 100 *µ*L metformin (1 mg/1 mL) + 100 *µ*L plant extract (1 mg/mL)

**Table 2:** Phytochemical constituents of sequentially extracted *P.  virgatus* fractions.

*P. virgatus* extracts	Tannins	Terpenoids	Phenols	Flavanoids	Protein	Glucose	Reducing sugar
*n*-hex	− −	− −	− −	− −	++	− −	− −
DCM	++	+	− −	− −	++	− −	− −
EtOAc	− −	++	− −	+++	++	+	+
Water	+++	+	++	+++	+++	− −	−
MeOH	++	++	++	+++	+	++	+

**Table 3:** Ferric reducing antioxidant potential and total phenol content of* P. virgatus* extracts. The data represents mean ± S.D. of six FRAP and three TPC experiments.

Extracts/reference	FRAP value (*μ*mol Fe(II)/g)	TPC (*μ*g GA/mg extract)
*n*-hex	0.442 ± 0.126	2.61 ± 0.017
DCM	0.924 ± 0.419	1.92 ± 0.001
EtOAc	1.255 ± 0.53	37.42 ± 0.010
MeOH	28.61 ± 0.2.184	176.68 ± 0.032
Water	5.69 ± 9.7	67.766 ± 0.029
Ascorbic acid	13.05 ± 3.131	

**Table 4:** IC_50_ values of *P. virgatus* against DPPH radicals, hydroxyl radicals, and *α*-amylase activity.

Activity	Plant extract/standard	IC_50 _(*µ*g/mL)
DPPH radical scavenging	*P. virgatus* (MeOH)	18.59 ± 0.515
	*P. virgatus* (water)	40.36 ± 2.35
	*P. virgatus* (EtOAc)	NS
	*P. virgatus* (DCM)	NS
	*P. virgatus* (*n*-hex)	NS
	Ascorbic acid	10.72 ± 0.33

Hydroxyl radical scavenging	*P. virgatus* (MeOH)	12.53 ± 2.38
	*P. virgatus* (water)	14.56 ± 0.389
	Mannitol	NS

*α*-Amylase inhibition	*P. virgatus* (MeOH)	33.20 ± 0.556
	Acarbose	76.88 ± 0.277

**Table 5:** Major constituents of *P. virgatus *methanol extract revealed via GC-MS analysis.

Peak	R.T	Compound	Molecular formula	Molecular weight	Area%
1	16.508	Benzenedicarboxylic acid (synonym: phthalic acid)	C_12_H_14_O_4_	222	7.1
2	17.005	2,4,5-Trimethoxy propenyl benzene (synonym: asarone)	C_12_H_16_O_3_	208	27
3	18.508	Tridecyl ester (synonym: acrylic acid)	C_16_H_30_O_2_	254	8.77
4	22.643	Hexadecanoic acid (synonym: palmitic acid)	C_17_H_34_O_2_	270	13.36
5	26.557	9,12-Octadecadienoic acid (synonym: linoleic acid)	C_19_H_34_O_2_	294	16.91
6	26.798	11-Octadecenoic acid	C_19_H_34_O_3_	294	22.29
7	27.517	6-Octadecynoic acid	C_19_H_34_O_4_	294	4.56

**Table 6:** Molecular docking results of pancreatic *α*-amylase with different compounds of *P. virgatus* methanol extract.

Compounds	Binding energy (kcal/mol)	Residues involved
Hexadecanoic acid	−4.58	TRP-59, TYR-62, GLN-63, LEU-162, LEU-165, ADP-197, ALA-198, LYS-200, HIS-201, ILE-235, ASP-300
Asarone	−5.21	LEU-162, ASP-197, ALA-198, LYS-200, HIS-201, GLU-233, VAL-234, ILE-235
Phthalic acid	−4.00	TYR-151, ALA-198, LYS-200, HIS-201, GLU-233, ILE-235
Acrylic acid	−5.19	TRP-59, TYR-62, GLN-63, LEU-162, LEU-165, LYS-200, HIA-201, GLU-233, VL-234, ILE-235
11-Octadecenoic acid	−5.55	TRP-59, TYR-62, LEU-162, LEU-165, LYS-200, HIS-201, GLU-233, VAL-234, ILE-235
9,12-Octadecadienoic acid	−6.11	TRP-59, TYR-62, GLN-63, LEU-162, LEU-165, ALA-198, LYS-200, HIS-201, GLU-233, ILE-235
6-Octadecynoic acid	−4.92	TRP-58, TRP-59, TYR-62, HIS-101, LEU-162, VAL-163, LEU-165, ASP-197, ALA-198, LYS-200, HIS-201, GLU-233, ILE-235, HIS-305
